# Indocyanine green fluorescence angiography in laparoscopic sigmoid and rectal cancer surgery: no reduction in anastomotic leakage but a lower incidence of anastomotic strictures. A prospective single-center study

**DOI:** 10.3389/fsurg.2026.1822071

**Published:** 2026-06-25

**Authors:** Solomiia Semeniv, Michał Pędziwiatr, Justyna Rymarowicz, Agnieszka Szpakowska, Michał Jurczak, Mateusz Putowski, Mateusz Rubinkiewicz

**Affiliations:** 1 Center for Innovative Medical Education, Jagiellonian University Medical College, Krakow, Poland; 2 Department of General, Oncologic, Metabolic and Emergency Surgery, University Hospital in Krakow, Kraków, Poland; 3 The 2nd Department of General Surgery, Jagiellonian University Medical College, Krakow, Poland; 4 Jagiellonian University Medical College, Krakow, Poland; 5 Clinical Department of Anesthesiology and Intensive Care, University Hospital in Krakow, Krakow, Poland; 6 Collegium Medicum, Jan Kochanowski University in Kielce, Kielce, Poland

**Keywords:** anastomosis, anastomotic leakage (AL), colorectal cancer, fluorescein angiography, indocyanine green (ICG), perfusion, surgical methods

## Abstract

**Introduction:**

Anastomotic leakage (AL) remains one of the most serious complications after colorectal cancer surgery and is closely linked to postoperative morbidity and poorer oncologic outcomes. Intraoperative indocyanine green fluorescence angiography (ICG-FA) has been introduced to allow real-time assessment of bowel perfusion; however, its role in reducing AL is still debated. Therefore, the primary aim of this study was to evaluate whether intraoperative ICG-FA, compared with standard visual assessment, reduces the rate of anastomotic leakage in patients undergoing laparoscopic sigmoid or rectal cancer resection. The secondary aims were to assess its impact on the incidence of anastomotic strictures, overall postoperative complications, reoperation rates, length of hospital stay, readmission, and 30-day mortality.

**Methods:**

We conducted a prospective single-center study with a retrospective historical control group, including consecutive patients undergoing laparoscopic sigmoid or rectal cancer resection. Patients who underwent intraoperative ICG-FA between October 2023 and January 2025 were compared with those undergoing similar procedures without fluorescence assessment. The primary endpoint was AL within 30 days. Secondary outcomes included anastomotic stricture within six months, postoperative complications, reoperation, length of hospital stay, readmission, and mortality.

**Results:**

A total of 113 patients were analyzed, including 34 in the ICG-FA group and 79 in the control group. The AL rate did not differ significantly between groups (14.7% vs. 12.7%, *p* = 0.768). No anastomotic strictures were observed in the ICG-FA group, while strictures occurred in 11.4% of patients in the control group (*p* = 0.050). Other postoperative outcomes, such as reoperation, length of stay, readmission, and 30-day mortality, were similar between groups.

**Conclusion:**

Intraoperative ICG-FA did not significantly reduce the rate of AL but was associated with a lower incidence of anastomotic strictures. These results imply that fluorescence-guided perfusion assessment may help improve anastomotic healing beyond just preventing clinically evident leakage. Larger prospective multicenter studies are needed to verify these findings.

**Clinical trial registration:**

https://clinicaltrials.gov/study/NCT07423130, ClinicalTrials.gov NCT07423130.

## 1 Introduction

Anastomotic leakage (AL) remains one of the most serious complications after colorectal surgery (1–3). Despite advances in minimally invasive techniques, perioperative management, and surgical standardization, the reported incidence of AL after sigmoid and rectal surgery ranges from nearly 3% to 23% (1, 4–7), and it is linked to increased postoperative complications, longer hospital stays, need for reintervention, stoma creation, decreased quality of life, and higher short-term mortality (8, 9). Additionally, AL can negatively impact long-term oncologic outcomes by delaying adjuvant therapy and increasing the risk of local recurrence, affecting disease-free survival and overall survival (7, 10–15).

The cause of AL is multifactorial, involving patient-related, disease-related, and intraoperative technique. Adequate perfusion of the bowel ends is essential for safe anastomotic healing (16–19). However, intraoperative assessment of bowel perfusion remains largely subjective and is usually based on visual inspection of tissue color, bleeding at the transection margin, mesenteric pulsation, and peristalsis. These indicators have limited sensitivity for detecting microcirculatory disturbances, and subclinical ischemia may go unnoticed. Such hidden perfusion deficits may contribute not only to clinically evident AL but also to impaired healing and late anastomotic complications (20, 21).

Indocyanine green fluorescence angiography (ICG-FA) has been introduced as an adjunctive, real-time tool for intraoperative assessment of bowel perfusion during colorectal surgery. After intravenous administration, ICG rapidly binds to plasma proteins, enabling visualization of tissue vascularization under near-infrared light (21–24). This technique allows surgeons to evaluate perfusion at the intended transection line and to adjust the level of resection or anastomosis if inadequate blood supply is detected. While early observational studies suggested a potential reduction in AL rates with ICG-guided perfusion assessment, subsequent randomized controlled trials and meta-analyses have yielded inconsistent results, with benefits reported mainly in specific subgroups (4, 23, 25–27). Importantly, most studies have primarily focused on AL as the main outcome, while other anastomosis-related complications, such as anastomotic stricture formation, have received limited attention. Previous studies have shown that anastomotic leakage is associated with an increased risk of subsequent stricture formation (28). The underlying mechanisms may involve local inflammation, fibrosis, and tissue remodeling at the anastomotic site. This suggests that factors affecting anastomotic healing, including perfusion, may influence outcomes beyond clinically evident leakage.

Given the ongoing uncertainty about the clinical value of ICG-FA in routine colorectal practice and the limited data on anastomotic stricture formation, more prospective real-world evidence is needed. Therefore, the goal of this prospective single-center study was to assess the impact of intraoperative ICG-FA on AL and other postoperative outcomes, including anastomotic stricture formation, in patients undergoing laparoscopic sigmoid and rectal cancer surgery.

## 2 Materials and methods

### 2.1 Study design

We conducted a prospective study with a retrospectively assembled historical control group at a tertiary-care hospital. Consecutive patients undergoing laparoscopic sigmoid and rectal cancer resection with intraoperative ICG-FA were prospectively enrolled between October 9, 2023, and January 15, 2025, at the Department of General, Oncological, Metabolic, and Emergency Surgery, University Hospital in Krakow. The control group included anonymized records of consecutive patients who underwent similar oncologic resections at the same institution between January 1, 2021, and December 31, 2024. Data were collected from retrospective chart reviews. The study protocol was registered at ClinicalTrials.gov (NCT07423130).

### 2.2 Eligibility criteria

Patients qualified for the ICG-FA group if they met the following inclusion criteria (1): age 18 years or older; (2) had a histologically confirmed or clinically suspected primary adenocarcinoma of the sigmoid colon or rectum; (3) presented with resectable disease (cT1–T4b, N0–3, M0-1; AJCC TNM, 8th edition); and (4) were eligible for curative laparoscopic resection, either as initial treatment or after neoadjuvant therapy. Exclusion criteria included: (1) unresectable distant metastases on preoperative imaging; (2) pregnancy or breastfeeding; and (3) allergy to iodine-containing compounds or prior adverse reactions to ICG. Withdrawal criteria included: (1) conversion to laparotomy; or (2) final pathology inconsistent with adenocarcinoma. Surgery was performed as clinically indicated in all such cases, but patients were excluded from the study.

### 2.3 Surgery

Combined oral and intravenous antibiotic prophylaxis was given. Mechanical bowel preparation was used only in patients scheduled for a diverting ileostomy; others received a preoperative enema. Oncologic resections were performed based on standard principles, including a lymphangiectomy around the inferior mesenteric artery, and mesocolic or mesorectal dissection along embryologic planes. Rectal cancers were treated with total mesorectal excision. Specimens were extracted through a Pfannenstiel incision. ICG powder (Verdye®, Diagnostic Green, Germany; 25 mg) was dissolved in sterile water to a final concentration of 2.5 mg/mL. A 10 mg dose was administered intravenously with a 20 mL saline bolus before transecting the proximal colon. Perfusion was evaluated using an Olympus Medical Imaging System or the Stryker 1688 AIM platform. Adequate perfusion was defined as the presence of visible serosal fluorescence within 60 s at the level of the mesocolic transection plane (29). When fluorescence at this site was delayed or absent, the transection point was moved proximally to the region showing satisfactory perfusion indicated by fluorescence.

To reduce potential sources of bias inherent in the non-randomized study design, several measures were implemented. Selection bias was minimized by enrolling consecutive patients who met predefined eligibility criteria and by conducting the study at a single tertiary referral center with consistent institutional indications for surgery. Performance bias was reduced through standardized perioperative management according to Enhanced Recovery After Surgery protocols and the use of homogeneous minimally invasive surgical techniques. To limit information bias, postoperative outcomes were defined using standardized, widely accepted criteria.

### 2.4 Data collection

Data were collected at three time points: preoperative, intraoperative, and postoperative. Baseline data included age, sex, body mass index (BMI), American Society of Anesthesiologists physical status (ASA) classification, comorbidities, history of prior surgeries, and preoperative staging. Neoadjuvant treatment regimens were recorded where applicable. Operative data covered procedure type, total operative time, intraoperative complications, and any adverse events related to ICG administration. Each patient in the study group underwent an ICG-related perfusion assessment. Postoperative outcomes included final histopathologic staging, anastomosis-specific complications, and overall complications graded by the Clavien-Dindo classification, length of hospital stay, 30-day readmission rate, and 30-day all-cause mortality (30, 31).

### 2.5 Outcomes

The primary endpoint was to determine whether intraoperative ICG-FA use decreases the rate of AL in patients undergoing laparoscopic resection for sigmoid and rectal cancer, compared to patients having laparoscopic surgery for the same conditions without ICG assessment. AL was defined per the International Study Group of Rectal Cancer as any defect in the integrity of the intestinal wall at the colorectal or coloanal anastomosis that results in communication between the intra- and extraluminal spaces, including nearby pelvic abscesses (32, 33). Each suspected case of AL required confirmation by contrast-enhanced computed tomography. We included AL in the study within 30 days of the procedure.

Secondary endpoints focused on perioperative safety outcomes associated with ICG-FA use. These included the incidence of other postoperative complications [such as anastomotic stricture, bleeding from the stapler line, anastomotic fistulas, deep or organ/space surgical site infection (SSI), ileus, urinary dysfunction], operation time, non-elective reoperations within 30 days after primary surgery, the length of postoperative hospital stay, hospital readmission within 30 days after discharge, and 30-day all-cause mortality. An anastomotic stricture was defined as a benign narrowing at the colorectal or coloanal anastomotic site resulting in a reduced luminal diameter, manifested by obstructive defecation symptoms, impaired endoscopic passage, or radiologic evidence of luminal narrowing, in the absence of recurrent malignant disease (34). To evaluate anastomotic stricture, patients were monitored for 6 months following the surgical procedure. An anastomotic fistula was defined as an abnormal connection between the bowel at the anastomotic site and another organ or body cavity, resulting from partial dehiscence or breakdown of the anastomosis (35). Anastomotic complications were included only if they were symptomatic and confirmed radiologically with computed tomography or magnetic resonance imaging, or endoscopically during flexible sigmoidoscopy or colonoscopy. A deep SSI was defined as an infection involving the fascial or muscle layers of the surgical incision occurring within 30 days after the procedure, confirmed by purulent drainage, wound dehiscence with clinical signs of infection, or radiologic, endoscopic, or intraoperative evidence of infection. An organ/space SSI was defined as an infection involving any intra-abdominal or pelvic space manipulated during surgery, excluding the incision, fascia, and muscle layers, confirmed by purulent drainage from a drain, positive cultures, or radiologic or operative findings consistent with infection (36, 37). Postoperative ileus was defined according to the most widely accepted criteria as the absence of flatus or stool passage, combined with intolerance to oral intake beyond postoperative day four (38). Urinary dysfunction was defined as the new onset of lower urinary tract symptoms after surgery, including urinary retention, voiding difficulties, urgency, or urinary incontinence (39).

### 2.6 Ethical approval

All procedures were conducted in accordance with the ethical standards of the Helsinki Declaration of 1975, as revised in Brazil in 2013 (40). The study received approval from the Research Ethics Board at Jagiellonian University Medical College (approval number: 1072.6120.59.2023). Written informed consent was obtained from all prospectively enrolled patients. Reporting adhered to the STROBE (Strengthening the Reporting of Observational Studies in Epidemiology) guidelines (41).

### 2.7 Statistical analysis

Data distribution was assessed using the Shapiro–Wilk test. Normally distributed variables were presented as mean ± SD, and non-normally distributed variables as median with interquartile range. Categorical variables were presented as counts and percentages. Between-group comparisons were performed using the independent-samples t-test or Mann–Whitney U test for continuous variables and the chi-square or Fisher's exact test for categorical variables. For the univariate analysis, each variable was assessed separately for its association with the occurrence of anastomotic leakage. As all analyzed variables were categorical, comparisons between groups were performed using the *χ*^2^ test or Fisher's exact test, as appropriate, depending on the expected cell counts. A two-sided *p*-value ≤ 0.050 was considered statistically significant. All analyses were performed using IBM SPSS Statistics version 29 (IBM Corp., Armonk, NY, USA).

## 3 Results

**Figure 1:**
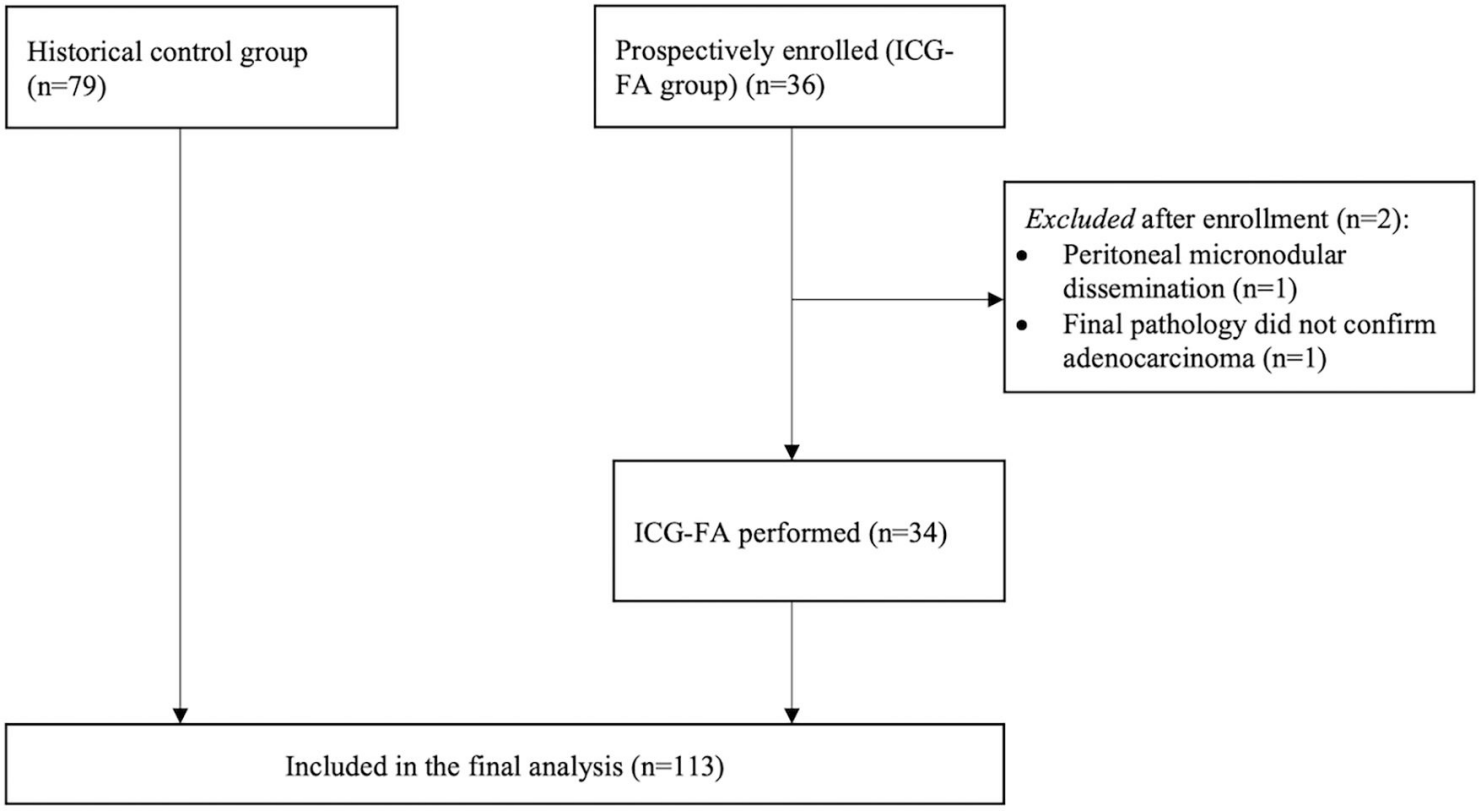
Study flow diagram. Flowchart of patient enrollment, exclusions based on predefined criteria, and final study population included in the comparative analysis between the prospective indocyanine green fluorescence angiography (ICG-FA) group and the historical control group.

A total of 113 patients were included in the final analysis, including 34 in the ICG-FA group and 79 in the control group (Figure 1).

### 3.1 Patient and tumor characteristics

Baseline characteristics of both groups are summarized in Table 1. No significant differences were observed between the groups in demographic or clinical characteristics. The prevalence of comorbidities and the rate of prior abdominal surgery were similar. Neoadjuvant treatment regimens were similarly distributed, including chemoradiotherapy (*p* = 1.000), chemotherapy (*p* = 0.827), and radiotherapy (*p* = 0.837). Clinical tumor stage according to the American Joint Committee on Cancer (AJCC) did not differ significantly between groups (*p* = 0.128), and final histopathologic staging was also comparable (*p* = 0.247).

**Table 1:** Patient and tumor characteristics. .

Characteristic	ICG-FA group (*n* = 34)	Control group (*n* = 79)	*p*-value
Sex, *n* (%)			
Male	19 (55.9)	51 (64.6)	0.405
Female	15 (44.1)	28 (35.4)	
Age (years), mean ± SD	64.2 ± 10.0	65.6 ± 10.0	0.478
BMI (kg/m^2^), mean ± SD	27.2 ± 5.1	27.6 ± 4.6	0.658
Comorbidities, *n* (%)			
Hypertension	21 (61.8)	49 (62.0)	1.000
Diabetes mellitus	8 (23.5)	19 (24.1)	1.000
Cardiac dysfunction	13 (38.2)	20 (25.3)	0.182
Pulmonary dysfunction	1 (2.9)	6 (7.6)	0.673
ASA class, *n* (%)			
I	0	4 (5.1)	0.252
II	11 (32.4)	30 (38.0)	
III	23 (67.6)	42 (53.2)	
IV	0	3 (3.8)	
Previous abdominal surgery, *n* (%)	15 (44.1)	40 (50.6)	0.546
Neoadjuvant therapy, *n* (%)			
Chemoradiotherapy	10 (29.4)	25 (31.6)	1.000
Chemotherapy	10 (29.1)	26 (32.9)	0.827
Radiotherapy	16 (47.1)	34 (43.0)	0.837
Tumor stage (AJCC) clinical, *n* (%)			
I	10 (29.4)	12 (15.2)	0.128
II	3 (8.8)	20 (25.3)	
III	19 (55.9)	43 (54.4)	
IV	2 (5.9)	4 (5.1)	
Tumor stage (AJCC) HP, *n* (%)			
I	13 (38.2)	18 (22.8)	0.247
II	6 (17.6)	21 (26.6)	
III	7 (20.6)	23 (34.2)	
IV	2 (5.9)	5 (6.3)	
CR	6 (17.6)	8 (10.1)	

ICG-FA, indocyanine green fluorescence angiography; SD, standard deviation; BMI, body mass index; ASA, American Society of Anesthesiologists class; AJCC, American Joint Committee on Cancer; HP, histopathological, CR, complete response.

### 3.2 Operative details and postoperative outcomes

The distribution of surgical procedures was similar between the groups, with low anterior resection (LAR) being the most common operation in both the ICG-FA group (44.1%) and the control group (44.3%) (*p* = 1.000). Median operative time was slightly longer in the ICG-FA group [235 min (IQR 188–257)] than in the control group [225 min (IQR 160–275)], although the difference was not statistically significant (*p* = 0.243). The AL rate was 14.7% in the ICG-FA group and 12.7% in the control group (*p* = 0.768). Rates of anastomotic bleeding and fistulas were comparable, whereas anastomotic strictures occurred only in the control group with a borderline statistically significant difference (11.4% vs. 0%; *p* = 0.050). Among patients with anastomotic stricture (*n* = 9), the majority were male (66.7%), underwent low anterior resection (55.6%), received neoadjuvant therapy (66.7%), and had a diverting ileostomy (55.6%), while clinically evident anastomotic leakage was observed in 44.4% of cases (Table 2). Deep and organ/space SSIs were reported in 8.8% of the ICG-FA group and 16.5% of the control group (*p* = 0.384). Other postoperative complications, including ileus (2.9% vs. 7.6%; *p* = 0.673) and urinary dysfunction (8.8% vs. 6.3%; *p* = 0.695), did not differ significantly. Reoperation within 30 days was required in 14.7% of patients in the ICG-FA group (5/34) and 11.4% of controls (9/79), with no significant difference between groups (*p* = 0.756). In the ICG = FA group, reoperations were performed mainly for AL (*n* = 3), while single cases were related to suspected leakage without confirmation and trocar-site omental protrusion. In the control group, reoperations were most commonly due to AL (*n* = 5), followed by early ileostomy closure due to poor tolerance (*n* = 2), pelvic bleeding (*n* = 1), and colonic perforation (*n* = 1). Length of postoperative hospital stay and 30-day readmission rates were likewise similar. No deaths occurred in either group within 30 days after surgery. All detailed results are presented in Table 3.

**Table 2:** Clinical and pathological characteristics of patients with anastomotic stricture.

Patient	Sex	Age (years)	Operation type	Ileostomy	pTNM (AJCC)	Neoadjuvant therapy	Clinical AL
1	M	63	ISR	Yes	T3N0M0R0LV0	CTH + RTH	Yes
2	M	71	ISR	Yes	T3N0M0R0LV0	RTH	Yes
3	M	69	LAR	No	T3N0M0R0LV0	No	Yes
4	F	74	LAR	Yes	T1N0M0R0LV0	CTH + RTH	No
5	F	41	LAR	Yes	CR	CTH + RTH	No
6	M	78	HAR	No	T1bN0M0R0LV0	No	No
7	M	75	LAR	No	TisN0M0R0LV0	RTH	Yes
8	M	52	ISR	Yes	T3N1M0R0LV0	CTH + RTH	No
9	F	67	LAR	No	T1N0M0R0LV0	No	No

AL, anastomotic leakage; HAR, high anterior resection; LAR, low anterior resection; ISR, intersphincteric resection; pTNM, pathological tumor–node–metastasis classification according to the American Joint Committee on Cancer (AJCC) staging system; CTH, chemotherapy; RTH, radiotherapy.

**Table 3:** Operative details and postoperative outcomes.

Characteristic	ICG-FA group (*n* = 34)	Control group (*n* = 79)	*p*-value
Surgical procedure, *n* (%)			
Sigmoidectomy	4 (11.8)	4 (5.1)	0.239
HAR	13 (38.2)	36 (45.6)	0.538
LAR	15 (44.1)	35 (44.3)	1.000
ISR	2 (5.9)	6 (7.6)	1.000
Protective ileostomy, *n* (%)	13 (38.2)	31 (39.2)	1.000
Operation time (min), mean ± SD	241 ± 71	226 ± 86	0.378
Complications, *n* (%)			
Anastomotic leakage	5 (14.7)	10 (12.7)	0.768
Bleeding from stapler line	1 (2.9)	4 (5.1)	1.000
Strictures	0	9 (11.4)	0.050
Fistulas	1 (2.9)	1 (1.3)	0.513
Deep/ organ space SSI	3 (8.8)	13 (16.5)	0.384
Ileus	1 (2.9)	6 (7.6)	0.673
Urinary dysfunction	3 (8.8)	5 (6.3)	0.695
Clavien-Dindo classification, *n* (%)			
I	23 (67.6)	52 (65.8)	0.283
II	3 (8.8)	12 (15.2)	
IIIa	2 (5.9)	9 (11.4)	
IIIb	5 (14.7)	6 (7.6)	
IVa	1 (2.9)	0	
V	0	0	
Reoperation within postoperative 30 days, mean ± SD	5 ± 14.7	9 ± 11.4	0.756
Length of postoperative hospital stay (days), mean ± SD	7.4 ± 10.4	5.8 ± 5.6	0.302
Readmission within postoperative 30 days, mean ± SD	3 ± 8.8	11 ± 13.9	0.548
Mortality within postoperative 30 days, mean ± SD	0	0	

ICG-FA, indocyanine green fluorescence angiography; HAR, high anterior resection; LAR, low anterior resection; ISR, intersphincteric resection; SD, standard deviation; SSI, surgical site infection.

### 3.3 Anastomotic leak: patient features and risk factors

AL occurred in 15 patients (13.3% overall): 14.7% (5/34) in the ICG-FA group and 12.7% (10/79) in the control group (*p* = 0.768). Characteristics of patients who developed AL in both groups are summarized in Table 4. Patients with AL in the ICG-FA group were younger (mean age 54.8 ± 5.4 years) and had a higher BMI (29.8 ± 6.8 kg/m^2^) compared with those in the control group (mean age 65.3 ± 7.7 years; BMI 25.8 ± 3.3 kg/m^2^). Operative time was longer among patients who developed AL, particularly in the ICG-FA cohort (298 ± 105.7 min vs. 255.3 ± 95.5 min). Univariate analysis showed no significant link between AL and neoadjuvant therapy (*p* = 0.582), tumor stage (*p* = 0.778), type of surgery (*p* = 0.591), or the presence of a diverting ileostomy (*p* = 0.779). A non-significant trend toward higher leakage rates was observed in low anterior and intersphincteric resections compared with high anterior or sigmoid resections (*p* = 0.094). The use of ICG-FA did not significantly decrease the incidence of AL (*p* = 0.768). Detailed results are provided in Table 5.

**Table 4:** Characteristics of patients with anastomotic leakage.

Characteristic	ICG-FA group (*n* = 5)	Control group (*n* = 10)
Sex, *n* (%)		
Male	4 (80)	8 (80)
Female	1 (20)	2 (20)
Age (years), mean ± SD	54.8 ± 5.4	65.3 ± 7.7
BMI (kg/m^2^), mean ± SD	29.8 ± 6.8	25.8 ± 3.3
Operation time (min), mean ± SD	298 ± 105.7	255.3 ± 95.5
Neoadjuvant therapy, *n* (%)		
Chemoradiotherapy	2 (40)	3 (30)
Chemotherapy	2 (40)	3 (30)
Radiotherapy	3 (60)	5 (50)
Tumor stage (AJCC) HP, *n* (%)		
I	4 (80)	0
II	0	4 (40)
III	1 (20)	5 (50)
IV	0	
CR	0	1 (10)

ICG-FA, indocyanine green fluorescence angiography; SD, standard deviation; BMI, body mass index; ASA, American Society of Anesthesiologists class; AJCC, American Joint Committee on Cancer; HP, histopathological, CR, complete response.

**Table 5:** Univariate analysis of variables associated with the incidence of anastomotic leakage.

Characteristic	Category	AL (+) *n* = 15	*p*-value
Sex	Male	12/70	0.158
	Female	3/43	
Age (years)	< 70	12/77	0.380
	≥ 70	3/36	
BMI (kg/m^2^)	≤25	5/39	1.000
	>25	10/74	
Tumor stage (AJCC) clinical, *n* (%)	Stages I and II	5/45	0.778
	Stages III and IV	10/68	
Neoadjuvant therapy	Yes	8/51	0.582
	No	7/62	
Surgical procedure	LAR, ISR	11/57	0.094
	SE, HAR	4/56	
Surgical procedure	HAR, LAR, ISR	15/106	0.591
	SE	0/7	
Operation time (min)	≤230*	5/63	0.092
	>230*	10/50	
Diverting ileostomy	Yes	5/44	0.779
	No	10/69	
ICG-FA	Yes	5/34	0.768
	No	10/79	

ICG-FA, indocyanine green fluorescence angiography; AL, anastomotic leakage; BMI, body mass index; AJCC, American Joint Committee on Cancer staging system; HAR, high anterior resection; LAR, low anterior resection; ISR, intersphincteric resection; SE, sigmoidectomy; SD, standard deviation.

*The mean operative time for the entire ICG-FA group was 230 ± 82 min. Based on this value, operative time was dichotomized into ≤230 min and >230 min and included as a variable in the univariate analysis.

## 4 Discussion

In our study, the use of intraoperative ICG-FA does not decrease the rate of AL in patients undergoing laparoscopic resection for sigmoid and rectal cancer, compared to patients undergoing laparoscopic surgery for the same indications without ICG assessment. An increasing number of high-quality randomized trials and meta-analyses have evaluated the use of ICG-FA in colorectal surgery; however, despite growing evidence, the reported clinical benefits remain inconsistent. Although some subgroup analyses have suggested a potential benefit in selected patient populations, particularly in left-sided colorectal resections, these findings should be interpreted cautiously due to limited statistical power and borderline significance. Notably, the multicenter randomized EssentiAL trial, which analyzed data from 839 patients undergoing minimally invasive (laparoscopic, robotic, or transanal) sphincter-preserving rectal cancer surgery, showed a significantly lower rate of AL (grades A–C) in the ICG-FA group compared with standard assessment (7.6% vs. 11.8%; *p* = 0.041) (42) Additionally, several recent meta-analyses and original studies suggest that ICG-FA is linked to a significant decrease in anastomotic leak rates, especially in mid-, low-, and ultra-low rectal resections, and may improve intraoperative decision-making and perioperative outcomes (22, 23, 26, 43–47). Consistent with our findings, the AVOID trial did not demonstrate a statistically significant reduction in the overall 30-day AL rate with ICG fluorescence imaging (6% vs. 9%, *p* = 0.23). However, a potential reduction was observed in left-sided colorectal resections, particularly in a *post hoc* analysis of rectosigmoid procedures, where ICG guidance was associated with a lower 90-day leakage rate (9% vs. 15%; RR 0.58, 95% CI 0.34–1.00, *p* = 0.045). The study includes both robotic and laparoscopic resections and covers malignant and benign diseases. These findings may suggests that the potential benefit of ICG could be more pronounced in higher-risk anatomical and physiological subgroups (25). Similarly, the ICG-COLORAL randomized trial reported that routine use of ICG fluorescence imaging did not significantly reduce the overall AL rate in laparoscopic colorectal surgery (5.8% with ICG vs. 7.9% without ICG, *p* = 0.16). However, a trend toward benefit was observed in left-sided resections (5.2% in the ICG-FA group vs. 9.5% in controls; OR 0.55, 95% CI 0.29–1.05) (48). Our findings did not confirm such benefits in patients undergoing rectosigmoid surgery in a single-center setting. Therefore, direct comparisons between these trials and our results should be done with caution, as the studied populations differ significantly in surgical approach, disease indication, and anastomotic risk profile.

Regarding perioperative safety, ICG-FA use was not associated with increased postoperative morbidity in our cohort. Length of hospital stay, reoperation rates, 30-day readmission, and short-term mortality were similar between groups. A recent meta-analysis including 6,877 patients demonstrated that ICG-FA significantly reduced total postoperative complications (OR 0.79, 95% CI 0.66–0.95, *p* = 0.01) and the need for reoperation (OR 0.33, 95% CI 0.17–0.65, *p* < 0.05), while also shortening postoperative hospital stay (mean difference 0.91 days, 95% CI 0.41–1.41, *p* < 0.05). The reduction in overall morbidity was accompanied by a lower incidence of anastomotic leakage (OR 0.50, 95% CI 0.42–0.61, *p* < 0.05). However, no significant differences were observed in 30-day readmission or mortality rates (23). Conversely, a systematic review including 3,231 patients demonstrated a significantly lower mean AL rate in the ICG-FA group compared with controls (5.16% vs. 12.16%, *p* < 0.01). No statistically significant differences were observed in reoperation rates (10.91% vs. 10.26%, *p* = 0.96), mortality (0.55% vs. 0.51%, *p* = 0.99), postoperative ileus (6.96% vs. 6.18%, *p* = 0.94), or wound infection rates (2.34% vs. 2.24%, *p* = 0.98). These findings support the overall safety of fluorescence-guided perfusion assessment during the perioperative period (22).

We observed a borderline statistically significant reduction in anastomotic stricture rates in the ICG-FA group. To date, no studies have directly compared the incidence of anastomotic stricture after colorectal surgery with and without ICG-FA, as the existing literature has focused mainly on AL and overall postoperative morbidity (25, 27, 42, 48, 49) Current evidence suggests that impaired anastomotic healing, driven by inadequate perfusion and microischemia, may predispose to subclinical AL, which in turn promotes persistent local inflammation and fibrosis at the anastomotic site (50, 51). Both clinically overt and subclinical leaks have been associated with an increased risk of subsequent stricture formation, with early leakage and the extent of the anastomotic defect identified as independent risk factors for stenosis (28, 52–55). Even leaks with minimal or unrecognized clinical manifestations may trigger chronic inflammation, tissue remodeling, and fibrotic healing, ultimately resulting in anastomotic narrowing (34). By enabling real-time assessment of bowel perfusion, ICG-FA may help identify and mitigate microischemia at the anastomotic site, thereby potentially limiting downstream processes that contribute to stricture formation. Nevertheless, despite a plausible pathophysiological rationale, this finding should be interpreted with caution, and larger prospective studies are required to confirm the observed association and its clinical relevance.

The heterogeneity of currently available results suggests that the effectiveness of fluorescence-guided perfusion assessment may be influenced by multiple patient- and procedure-related factors. Therefore, future research should focus on identifying anatomical and physiological variables that may affect the benefit of ICG-FA, particularly in left-sided colorectal resections. Variability in colonic vascular anatomy and the burden of atherosclerotic disease, as assessed on preoperative imaging, may contribute to differences in perfusion and anastomotic healing (56, 57). In addition, quantitative fluorescence assessment represents a promising direction for future studies. Compared with subjective visual interpretation, quantitative ICG analysis may provide a more objective and reproducible evaluation of tissue perfusion and could improve standardization of intraoperative decision-making (58, 59).

This study has several limitations. First, the non-randomized design and use of a historical control group introduce potential selection and temporal bias. Second, the sample size was relatively small, and the study was not powered to detect small differences in leakage rates. Third, multivariable adjustment was not performed due to the limited sample size and low number of events, as such analysis could have resulted in model overfitting and unreliable estimates. Nevertheless, the multifactorial nature of anastomotic leakage should be acknowledged, and residual confounding cannot be excluded. Finally, intraoperative variables that influence perfusion, such as the vessel ligation strategy, were not consistently documented, but according to the literature, they could be useful (60). Despite these limitations, our study provides prospective real-world data reflecting routine clinical practice in a tertiary referral center. The standardized surgical technique and homogeneous minimally invasive approach strengthen internal consistency.

## 5 Conclusion

Intraoperative ICG-FA did not significantly reduce the AL rate but was associated with a lower incidence of anastomotic strictures. The technique may support intraoperative decision-making in colorectal surgery; however, it should be viewed as an adjunct rather than a replacement for standard surgical judgment. Further prospective, multicenter randomized studies are needed to better define its role in preventing anastomotic leaks and improving long-term outcomes, particularly in relation to vascular anatomical variability and the development of quantitative perfusion assessment methods.

## Data Availability

The raw data supporting the conclusions of this article will be made available by the authors, without undue reservation.
